# Genetic potential for biofilm formation of clinical strains of Pseudomonas aeruginosa

**DOI:** 10.18699/vjgb-25-62

**Published:** 2025-07

**Authors:** U.М. Nemchenko, N.L. Belkova, E.S. Klimenko, N.E. Smurova, R.E. Zugeeva, V.V. Sinkov, E.D. Savilov

**Affiliations:** Institute of Epidemiology and Microbiology, Scientific Center for Family Health and Human Reproduction Problems, Irkutsk, Russia; Institute of Epidemiology and Microbiology, Scientific Center for Family Health and Human Reproduction Problems, Irkutsk, Russia; Institute of Epidemiology and Microbiology, Scientific Center for Family Health and Human Reproduction Problems, Irkutsk, Russia; Institute of Epidemiology and Microbiology, Scientific Center for Family Health and Human Reproduction Problems, Irkutsk, Russia; Institute of Epidemiology and Microbiology, Scientific Center for Family Health and Human Reproduction Problems, Irkutsk, Russia; Institute of Epidemiology and Microbiology, Scientific Center for Family Health and Human Reproduction Problems, Irkutsk, Russia; Institute of Epidemiology and Microbiology, Scientific Center for Family Health and Human Reproduction Problems, Irkutsk, Russia

**Keywords:** Pseudomonas aeruginosa, cystic fibrosis, biofilms, whole genome sequencing, signaling pathways, Pseudomonas aeruginosa, муковисцидоз, биопленки, полногеномное секвенирование, сигнальные пути

## Abstract

Pseudomonas aeruginosa is one of the leading causes of nosocomial respiratory tract infections and plays an important role in lower respiratory tract infection in patients with cystic fibrosis (CF). Biofilms, which are organized cell clusters, ensure the survival of microorganisms in unfavorable environmental conditions and contribute to the chronicity of infection and the formation of persistent forms. The aim of this study was to determine the phenotypic ability and genetic potential for biofilm formation in clinical strains of P. aeruginosa persisting in patients with CF against the background of constant intake of antimicrobial drugs. Bacteriological, genetic, and bioinformatic methods were used to characterize five P. aeruginosa strains obtained from patients with CF. Phenotypically, all strains were classified as moderately biofilm-forming, while the biofilm formation coefficient varied from 2.10 to 3.15. Analysis of draft genomes revealed differences in the representation of some genes or individual loci of three of the four known signaling pathways (cAMP/Vfr, Gac/Rsm, and c-di-GMP) that have been described in P. aeruginosa genomes and are related to the regulation of biofilm formation. In addition, differences in the representation of genes such as frzE, tcpE, and rcsC are shown. Of undoubted interest is the analysis of genes such as pppA, icmF, clpV1, trpE, trpG, and stp1, which are used for extended multilocus typing PubMLST and differed in the structure of loci in all analyzed strains. These genes can be used to identify clinical strains of P. aeruginosa and to characterize their biofilm-forming properties. Thus, genes potentially participating in both biofilm formation and regulation have been characterized in the genomes of clinical P. aeruginosa strains that persist for a long time in patients receiving continuous antibiotic therapy. Characterization of the genetic potential for biofilm formation makes it possible to search for reliable genetic markers of this process in order to monitor the evolution of the pathogen as a result of long-term persistence in the host organism.

## Abstract (ru)

Бактерии вида Pseudomonas aeruginosa являются одной из ведущих причин нозокомиальных инфекций дыхательного тракта и играют важную роль в инфицировании нижних дыхательных путей у больных муковисцидозом (МВ). Биопленки, представляя собой организованные скопления клеток, обеспечивают выживаемость микроорганизмов в неблагоприятных условиях окружающей среды и способствуют хронизации инфекции и формированию персистирующих форм. Целью работы стало определение фенотипической способности и генетического потенциала к биопленкообразованию у клинических штаммов P. aeruginosa, персистирующих у пациентов с МВ на фоне постоянного приема антимикробных препаратов. Для характеристики пяти штаммов P. aeruginosa, полученных от пациентов с МВ, в работе применены бактериологические, молекулярно-генетические и биоинформатические методы. Фенотипически все штаммы отнесены к умеренно-образующим биопленку, при этом коэффициент биопленкообразования варьировал от 2.10 до 3.15. Анализ драфт-геномов выявил различия в представленности некоторых генов или отдельных локусов трех из четырех известных сигнальных путей, цАМФ/Vfr, Gac/Rsm и c-di-GMP, которые описаны в геномах P. aeruginosa и имеют отношение к регуляции образования биопленок. Дополнительно показаны отличия в представленности таких генов, как frzE, tcpE и rcsC. Несомненно интересен анализ генов pppA, icmF, clpV1, trpE, trpG и stp1, которые используются для расширенного мультилокусного типирования PubMLST и различаются по структуре локусов у всех проанализированных штаммов. Эти гены потенциально могут быть применены для типирования клинических штаммов P. aeruginosa с целью характеристики их биопленкообразующих свойств. Таким образом, в геномах клинических штаммов P. aeruginosa, длительно персистирующих у пациентов на фоне постоянного получения антибиотикотерапии, охарактеризованы гены, которые потенциально могут участвовать как в процессе биопленкообразования, так и в его регуляции. Характеристика генетического потенциала к образованию биопленок дает возможность поиска надежных генетических маркеров этого процесса для мониторинга эволюции возбудителя в результате длительной персистенции в организме хозяина.

## Introduction

Pseudomonas aeruginosa is one of the leading causes of nosocomial respiratory tract infections and plays an important role in lower respiratory tract infections in patients with cystic fibrosis (CF) (Parkins et al., 2018; Shaginyan et al., 2019). Surface colonization and subsequent biofilm formation and development provide numerous advantages for infectious agents. Biofilms, which are organized clusters of cells enclosed in a polysaccharide matrix and protected from adverse environmental conditions, including antimicrobials, disinfectants, and antiseptics, ensure microorganism survival. Such structural organization contributes to the increased heterogeneity of the bacterial population and the selection of cells that counteract damaging effects by acquiring and accumulating genetic mutations. Therefore, biofilm-forming microorganisms significantly contribute to the chronicity of infection and the formation of persistent forms (Penesyan et al., 2021).

Considering the pathogenetic potential of P. aeruginosa in the biofilm state in patients with CF, it should be noted that bacterial cells are tolerant not only to antibiotics, disinfectants, and antiseptics, but also to factors of the innate and adaptive defense system of the body (Jurado-Martín et al., 2021; Fernández-Billón et al., 2023). In response to anaerobic conditions, competition for resources, high concentrations of antibiotics, and immune responses of the body, such as neutrophil attacks in the lungs in CF, P. aeruginosa undergoes microevolution, acquiring spontaneous mutations that lead to the selection of cells that better survive long-term colonization (Winstanley et al., 2016; Jurado-Martín et al., 2021). Experimental studies have shown the ability of P. aeruginosa to form biofilm structures in the sputum of patients with CF (Bjarnsholt et al., 2009), which is a decisive factor for survival and tolerance to antibiotics, and the inability to completely eliminate bacteria is directly associated with the chronicity of the infection (Elfadadny et al., 2024). Understanding the mechanisms of adaptation and evolution of the pathogen during chronic respiratory infections in patients with CF may help discover new treatment methods for P. aeruginosa infections.

Modern sequencing technologies allow us to analyze the complete genomes of opportunistic microorganisms not only for their typing but also for identifying molecular markers that are potentially significant for the infectious agent. Currently, a map of the main signaling pathways characterized in the P. aeruginosa genomes and related to the regulation of biofilm formation has been created on the KEGG PATHWAY Database platform (PATHWAY: ko02025; https://www.genome. jp/kegg-bin/show_pathway?ko02025). The ko02025 map contains information on 90 loci included in four main signaling pathways: the cAMP/Vfr pathway, the quorum sensing (QS) system, the Gac/Rsm pathway, and the c-di-GMP signaling pathway.

The aim of this study was to determine the phenotypic ability and genetic potential for biofilm formation in clinical strains of P. aeruginosa persisting in patients with CF against the background of constant use of antimicrobial drugs.

## Materials and methods

The objects of the study were five clinical strains of P. aeruginosa from the working collection of the microbiome and microecology laboratory of the Institute of Epidemiology and Microbiology, Scientific Center for Family Health and Human Reproduction Problems. The strains were isolated from the sputum of patients with CF who were treated at Ivano- Matreninskaya City Children’s Clinical Hospital (Irkutsk, Russia) and who received long-term antibiotic therapy. The strains were identified using morpho-biochemical tests and were confirmed using mass spectrometry (Nemchenko et al., 2022). Sensitivity to AMPs was determined according to EUCAST criteria (Nemchenko et al., 2024).

The ability of the strains to form biofilms (BF) was studied using the G.A. O’Toole plate method (O’Toole, 2011), with our own modifications (Nemchenko et al., 2020; Sitnikova et al., 2022). Briefly, the ability of cultures to form BFs was determined using a 96-well sterile flat-bottomed plastic immunological plate. Daily bacterial culture was standardized in sterile meat-peptone broth (MPB) to 1 × 106 CFU/ml. The culture suspension and control were inoculated with 150 μl per well of the plate into four replicates. Sterile MPB served as the background control. The plate was incubated in a dryair thermostat for 18–20 h at 37 °C. Biofilms were stained using a modified G.A. O’Toole method: planktonic cells were removed by pipetting, the plate was washed three times with sterile saline, dried for 10–15 min without a lid, and the biofilms were stained with 1 % gentian violet, followed by alcohol extraction according to the method (O’Toole, 2011). The biomass of the formed films was estimated from the optical density (OD) of the gentian violet dye extracts at 492 nm (STAT FAX®4300 spectrophotometer, USA). The biofilm formation coefficient (BFC) was calculated as the A492exp/ A492control ratio. Strains with BFC values ≤ 2 units were considered to be weakly BF-forming; those with a BFC of 2–3.99 had a moderate ability to form BFs (Nemchenko et al., 2020; Grigorova et al., 2021).

Whole-genome sequencing. Genomic DNA was isolated using a Quick-DNA Fungal/Bacterial Miniprep Kit (Zymo Research, USA). Whole-genome sequencing of strains was performed on Illumina NextSeq 550 equipment using the Illumina ® DNA Prep Tagmentation, IDT® for Illumina® DNA/ RNA UD Indexes Set Tagmentation, and NextSeq 500/550 High Output Kit v.2.5 (300 Cycles) library preparation reagent kits according to the manufacturer’s recommendations.

Bioinformatics analysis. Genome assembly was performed using the SPAde sv.3.11.1 program (Bankevich et al., 2012). Contig alignment and orientation correction were performed using MAUVE 2.4.0 (Rissman et al., 2009) and the P. aeruginosa PAO1 reference genome (GenBank AE004091.2) (Table 1). Functional annotation was performed using Prokka 1.14.6 (Seemann, 2014). The genes involved in biofilm formation were identified using the KEGG (Kanehisa et al., 2022) and PubMLST (Jolley et al., 2018) databases. The complete genomes of the strains obtained in this study were deposited in the NCBI database project PRJNA1026796.

**Table 1.**
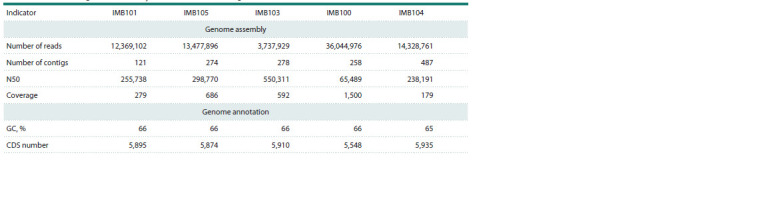
Brief results of genome assembly and annotation of P. aeruginosa strains

The study was conducted using equipment from the Center for collective use “Center for the development of progressive personalized health technologies”, and “Collection of human microbiota of the Irkutsk region” of the Institute of Epidemiology and Microbiology, Scientific Center for Family Health and Human Reproduction Problems (Irkutsk).

## Results

Five clinical strains of P. aeruginosa isolated from patients with CF who had received long-term antibiotic therapy were analyzed in the study. Phenotypically, all strains were classified as moderately biofilm-forming, with the BFC varying from 2.10 to 3.15 (Table 2). The minimum BFC value was determined for strain IMB101, and the maximum values were 3.11 and 3.15 for IMB100 and IMB104, respectively.

**Table 2.**
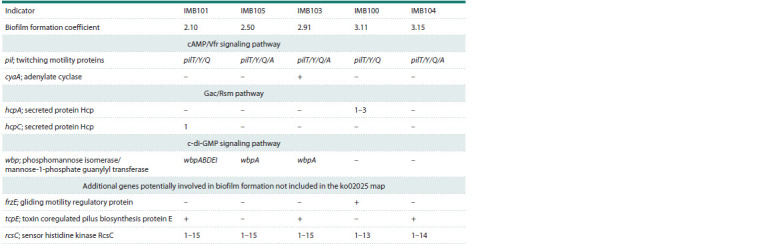
Phenotypic characteristics and genetic potential of P. aeruginosa strains for biofilm formation Notе. Numbers indicate the number of variants of the hcpA, hcpC, and rcsC genes according to genome annotation using Prokka 1.14.6 (Seemann, 2014).

Whole-genome sequencing was performed for all strains. The primary objective of this study was to identify genes that could participate in biofilm formation and its regulation. The ko02025 map (KEGG PATHWAY Database) was used for routine search. Additionally, we analyzed the loci used in typing P. aeruginosa strains on the PubMLST platform (Jolley et al., 2018) and manually searched for genes that had previously been shown to participate in the process of biofilm formation or its regulation (Thelin, Taylor, 1996; Kearns, Shimkets, 1998; Wall et al., 2018).

We showed that all tested genes are localized in chromosomal DNA. Differences between the genomes of the studied strains were found in the presence or absence of some genes or in the representation of loci of three signaling pathways: cAMP/Vfr, Gac/Rsm, and c-di-GMP (Table 2). An additional search for genes involved in biofilm formation revealed differences in the presence or absence of genes primarily involved in regulatory processes: frzE (gliding motility regulatory protein), tcpE (toxin coregulated pilus biosynthesis protein E), and rcsC (sensor histidine kinase RcsC) (Table 2). Of greatest interest are genes that not only participate in signaling pathways according to the ko02025 map (KEGG PATHWAY Database) but are also used for extended multilocus typing PubMLST (Table 3).

**Table 3.**
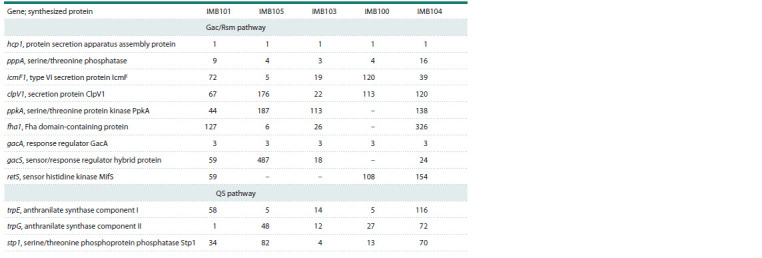
Description of loci identified in the genomes of the studied P. aeruginosa strains for genes used for extended MLST typing (PubMLST) and included in the ko02025 map

The hcp1 and gacA genes were completely identical in the genomes of the studied strains. Genes such as ppkA, fha1, and gacS were not identified in the IMB100 strain, which showed a fairly high biofilm formation coefficient (3.11). Note that retS was absent in the genomes of the IMB103 and IMB105 strains, which had biofilm formation coefficients of 2.91 and 2.50, respectively. Of undoubted interest is the analysis of genes such as pppA, icmF, clpV1, trpE, trpG, and stp1, which differed in the structure of loci in all the analyzed strains. These genes can be used to identify clinical strains of P. aeruginosa and to characterize their biofilm-forming properties.

## Discussion

P. aeruginosa is an opportunistic pathogen that causes infections in immunocompromised or CF patients. P. aeruginosa infection in CF patients occurs as a mild acute infection that subsequently progresses to chronic respiratory disease. It has been suggested that two distinct, mutually exclusive sets of virulence factors are associated with the two stages of infection (Brencic, Lory, 2009). The type III secretion system (T3SS) and type IV pili genes are thought to be associated with acute disease, whereas the type VI secretion system (T6SS) HSI-I and biofilm formation are important during chronic infection (Deretic et al., 1995; Brencic, Lory, 2009). There is currently an active search for genes, the expression of which differs according to the lifestyles of bacterial pathogens and which may be biomarkers of the transition from acute to chronic infection and vice versa (Cao et al., 2023).

In this study, we analyzed the phenotypic and genetic properties of P. aeruginosa strains isolated from patients with CF receiving continuous antibacterial therapy. All strains were defined as moderate biofilm-forming, but their genomes showed differences in the presence/absence of some genes or in the loci of signaling pathways that were characterized in the genomes of clinical P. aeruginosa strains and were related to both biofilm formation and regulation of this process. The identification of genetic markers of phenotypes associated with biofilm structure formation is of undoubted interest These genes may be used for extended multilocus typing PubMLST and may be responsible for individual stages of biofilm formation or regulation. In our studies, we identified 12 such genes, 9 of which belong to the Gac/Rsm signaling pathway, and 3 – to the QS pathway

The two-component GacS/GacA system stimulates the expression of two small regulatory RNAs, RsmY and RsmZ, which in turn regulate the translational repressor RsmA. Members of the RsmA/CsrA family have been identified in the genomes of many Gram-negative bacteria, including P. aeru-ginosa, P. fluorescens, Escherichia coli, and some species of Salmonella, Legionella, Proteus, Helicobacter, and Erwinia, where they have been implicated in the regulation of phenotypes such as virulence, motility, QS systems, and stress response (Brencic, Lory, 2009).

QS systems are a form of bacterial intercellular communication used by many species to determine population density and coordinate gene expression (Coggan, Wolfgang, 2012). QS is achieved by producing autoinducer signaling molecules so that an increase in bacterial population density leads to their accumulation. Once a threshold concentration is reached, autoinducers bind their cognate receptors, which directly or indirectly activate gene expression. Three QS systems are encoded in the genomes of P. aeruginosa: two N-acyl-homoserine lactone (AHL)-based and a 2-alkyl-4-quinolone (AQ)-based signaling system. These three QS systems are involved in the regulation of virulence factor production, biofilm maturation, and motility phenotypes (Coggan, Wolfgang, 2012).

It should be noted that studies of transcriptional profiles of different clinical P. aeruginosa strains grown under planktonic and biofilm conditions showed that transcriptional profiles detected under planktonic growth conditions were quite similar, and more divergent transcriptional profiles were recorded when isolates were grown under biofilm conditions (Thöming et al., 2020). The model experiments showed that different groups of clinical isolates follow parallel evolutionary pathways and produce similar phenotypes. This convergence of organismal phenotypes was observed for a variety of traits, including the formation of different biofilm structures characterized by specific transcriptional signatures, as well as virulence and motility phenotypes (Thöming et al., 2020).

It can be assumed that, despite the different sequence types identified in patients with CF, the transition to a persistent form during chronic P. aeruginosa infection will not simply stimulate the expression of certain genes to create a certain pathogen phenotype but will also form the corresponding genotype, realizing the potential of genetic heterogeneity of the bacterial population. It should also be noted that genes that participate in biofilm formation or regulation of this process (according to the ko02025 map) are used for extended multilocus typing of PubMLST and can be used to type clinical strains of P. aeruginosa in order to characterize their biofilmforming properties

## Conclusion

In the genomes of clinical strains of P. aeruginosa that persist for a long time in patients with CF against the background of constant antibiotic therapy, genes that can potentially participate both in the process of biofilm formation and in its regulation have been characterized. Characterization of the genetic potential for biofilm formation makes it possible to search for reliable genetic markers of this process to monitor the evolution of the pathogen as a result of long-term persistence in the host organism.

## Conflict of interest

The authors declare no conflict of interest.
